# Potential Renal Damage Biomarkers in Alport Syndrome—A Review of the Literature

**DOI:** 10.3390/ijms23137276

**Published:** 2022-06-30

**Authors:** Ana Marta Gomes, Daniela Lopes, Clara Almeida, Sofia Santos, Jorge Malheiro, Irina Lousa, Alberto Caldas Afonso, Idalina Beirão

**Affiliations:** 1 Nephrology Department, Hospital Centre Vila Nova de Gaia/Espinho, 4434-502 Vila Nova de Gaia, Portugal; ampgomes@gmail.com (A.M.G.); bannyel@gmail.com (D.L.); clara.almeida24@gmail.com (C.A.); 2 UMIB—Unit for Multidiscisciplinary Research on Biomedicine, Department of Nephrology, Dialysis and Transplantation, ICBAS—School of Medicine and Biomedical Sciences, University of Porto, Rua de Jorge Viterbo Ferreira n.º 228, 4050-313 Porto, Portugal; sofia.fersantos@gmail.com (S.S.); jjorgemalheiro@gmail.com (J.M.); 3 ITR, Laboratory for Integrative and Translational Research in Population Health, 4050-313 Porto, Portugal; 4 Nephrology Department, University Hospital Centre of Porto (CHUP), 4099-001 Porto, Portugal; 5 UCIBIO/REQUIMTE, Laboratory of Biochemistry, Department of Biological Sciences, Faculty Pharmacy, University of Porto, 4050-313 Porto, Portugal; irina.filipa@hotmail.com; 6 Paediatrics Department, University Hospital Centre of Porto (CHUP), 4099-001 Porto, Portugal; caldasafonso.cmin@chporto.min-saude.pt; 7 European Rare Kidney Disease Centre (ERKNET)—Universitary Hospital Centre of Porto (CHUP), 4099-001 Porto, Portugal

**Keywords:** Alport syndrome, hereditary kidney disease, COL4A, biomarkers

## Abstract

Alport syndrome (AS) is the second most common cause of inherited chronic kidney disease. This disorder is caused by genetic variants on *COL4A3*, *COL4A4* and *COL4A5* genes. These genes encode the proteins that constitute collagen type IV of the glomerular basement membrane (GBM). The heterodimer COL4A3A4A5 constitutes the majority of the GBM, and it is essential for the normal function of the glomerular filtration barrier (GFB). Alterations in any of collagen type IV constituents cause disruption of the GMB structure, allowing leakage of red blood cells and albumin into the urine, and compromise the architecture of the GFB, inducing inflammation and fibrosis, thus resulting in kidney damage and loss of renal function. The advances in DNA sequencing technologies, such as next-generation sequencing, allow an accurate diagnose of AS. Due to the important risk of the development of progressive kidney disease in AS patients, which can be delayed or possibly prevented by timely initiation of therapy, an early diagnosis of this condition is mandatory. Conventional biomarkers such as albuminuria and serum creatinine increase relatively late in AS. A panel of biomarkers that might detect early renal damage, monitor therapy, and reflect the prognosis would have special interest in clinical practice. The aim of this systematic review is to summarize the biomarkers of renal damage in AS as described in the literature. We found that urinary Podocin and Vascular Endothelial Growth Factor A are important markers of podocyte injury. Urinary Epidermal Growth Factor has been related to tubular damage, interstitial fibrosis and rapid progression of the disease. Inflammatory markers such as Transforming Growth Factor Beta 1, High Motility Group Box 1 and Urinary Monocyte Chemoattractant Protein- 1 are also increased in AS and indicate a higher risk of kidney disease progression. Studies suggest that miRNA-21 is elevated when renal damage occurs. Novel techniques, such as proteomics and microRNAs, are promising.

## 1. Introduction

Alport syndrome (AS) is a hereditary kidney disease due to genetic variants of *COL4A3*, *COL4A4* and *COL4A5* genes [1]. These genes encode proteins forming a heterodimer—α3α4α5—that constitutes collagen type IV, the major component of the GBM. Mutations in these genes affect the synthesis, assembly, deposition or function (or a combination of these) of the collagen type IV molecules by podocytes in the kidney [2]. The dysfunctional GBM leads to hematuria and albuminuria and, consequently, to chronic inflammation and fibrosis [3].

Collagen type IV also exists in the cochlea (striae vascularis), cornea (Descemet’s and Bowamn’s membrane), lens capsule and retina (inner limiting membrane and Brunch’s membrane) [4]. Extra-renal manifestations, such as sensorineural deafness and ocular anomalies (corneal opacities, anterior lenticonous, fleck retinopathy, temporal retinal thinning, and more rarely, posterior polymorphous corneal dystrophy, giant macular hole and maculopathy), can also occur [4,5]. Rare manifestations include leiomyomas in the esophagus, tracheobronquial tree and female genitalia and are associated with contiguous gene deletions in *COL4A6* and *COL4A5*. Vascular abnormalities, such as aortic aneurysms, have also been reported [6].

AS is the second most common monogenic cause of CKD after autosomal dominant polycystic kidney disease [3]. Data from the Genomics England 100,000 Genomes Project predicted pathogenic *COL4A5* variants occurred in at least one in 2320 individuals, heterozygous *COL4A3* or *COL4A4* variants affected one in 106, and compound heterozygous *COL4A3* or *COL4A4* variants affected one in 88,866 [7].

**Figure 1:**
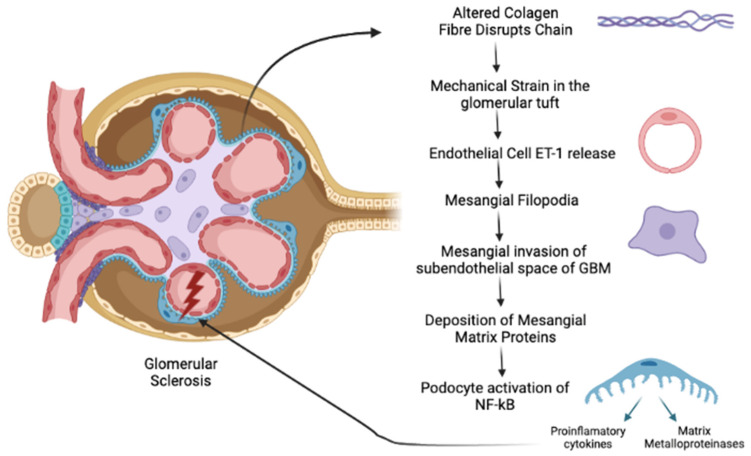
Pathogenic pathways of AS.

In AS, changes in GBM composition are the starting point of the pathogenic pathway that involves all three cell types of the glomerulus. The thinner GBM has fewer interchain disulfide crosslinks, which diminishes the elasticity of glomerular tufts; this makes them susceptible to mechanical forces of normal glomerular capillary flow, which has the highest capillary pressure in the human body [8,9,10]. One of the earliest events in AS is the significantly elevated expression of endothelin-1 (ET-1) in the endothelial cells, which seems to be related to an elevated biomechanical strain on glomerular capillary tufts. ET-1 elevation causes the activation of endothelin A receptors (ETARs), which in turn leads to downstream activation of CDC42, which results in mesangial filopodia. The mesangial filopodia progressively invade the subendothelial space of GBM, with deposition of mesangial matrix proteins, including laminin 211 [10,11]. Laminin 211 activates focal adhesion kinase (FAK) on podocytes, which activates NF-ĸB (Nuclear factor kappa B), resulting in elevated expression of pro-inflammatory cytokines as well as matrix metalloproteinases. In addition, laminin 211 contributes to altering GBM permeability and appears to be important to the progressive increase in proteinuria [10]. The deposition of mesangial matrix proteins in the GBM induces podocyte injury [10]. Progressive loss of podocytes causes glomerulosclerosis, which is associated with reduced glomerular blood perfusion to post-glomerular peritubular capillaries and reduced or absent glomerular filtrate, which together induce ischemic and inflammatory tubular cell injury, death, and peritubular inflammation and scarring [12]. The pathogenic pathways of AS are described in Figure 1.

The spectrum of kidney damage is broad and ranges from microscopic hematuria, to albuminuria and severe proteinuria, to progressive decline in glomerular filtration rate (GFR) and ESRD [1,13]. The loss of GFR can vary from very rapid, requiring kidney replacement therapy (KRT) in adolescence/early adulthood, to very slow, as in advanced age patients with normal kidney function [13], and is related to the pattern of genetic inheritance. 

Genetic inheritance patterns include X-linked (X-LAS), autosomal recessive (ARAS), autosomal dominant (ADAS) and digenic [1]. 

The *COL4A5* gene is located at chromosome X. Mutations on this gene severely affect male patients, who present the classic phenotype of kidney failure, sensorial deafness, and specific ocular lesions. Female patients can also be affected but present more attenuated clinical features [1].

ARAS occurs when both alleles of the gene present a genetic variant. Usually these patients have renal disease, deafness and ocular manifestations, and this pattern of inheritance is suggested when male and female patients are equally affected, when there is parental consanguinity or when the father or both parents of a male patient have microscopic hematuria [1]. 

ADAS occurs when one allele of *COL4A3* or *COL4A4* has a genetic variant. Clinical findings can include no symptoms, isolated hematuria (sometimes intermittent) [13], progressive proteinuria, and CKD that can lead to ESRD. Involvement of other organs is not frequent. Within the same family, clinical manifestations can be different due to incomplete penetrance and associated risk factors such as smoking, high blood pressure and levels of salt and animal protein intake [1]. ADAS patients progress more slowly to ESRD than X-LAS males and ARAS [14].

Digenic AS occurs when there are inherited pathogenic variants in *COL4A5* plus *COL4A3* or *COL4A5* or in *COL4A3* plus *COL4A4* [15,16,17]. For digenic variants affecting *COL4A5* plus *COL4A3* or *COL4A4*, clinical features depend on sex and the severity of both variants [15]. In the former situation, the two pathogenic variants in *COL4A3* and *COL4A4* may occur in the same (*in cis*) or opposite (*in trans*) chromosomes. There are limited data in these group of patients, especially concerning extra-renal features, but renal phenotype seems to be intermediate between ADAS an ARAS [15].

There are no specific light microscopy histological findings in AS, and a variety of combined features can be found, such as mild glomerular changes, mesangial proliferation and expansion, interstitial foam cells (when long-standing proteinuria occurs), focal segmental glomerulosclerosis (FSGS) and tubular atrophy and interstitial fibrosis. Moreover, cortical interstitial volume fraction and global sclerosis were found to be inversely correlated with creatinine clearance [18]. Electronic microscopy (EM) findings can range from thin basement membrane and GBM thickening to lamellation and splitting in GBM *lamina densa* [1,19]. Importantly, pathological findings may be apparent only as renal disease progresses, even in male X-LAS and ARAS patients. In female X-LAS and ADAS patients, typical electron microscopic lesions can be present only at later stages, and often only GBM thinning can be found [5]. Due to these findings, patients presenting with isolated microscopic hematuria and heterozygous variants in *COL4A3* and *COL4A4* have ADAS, and the diagnosis of thin basement membrane should not be used [1]. 

At present, with the recognition of different inheritance patterns and the broad spectrum of renal and extra-renal manifestations, genetic testing has been recommended to confirm the diagnosis of AS. In addition, genetic testing is more sensitive and specific than kidney biopsy, and it is currently considered the gold standard [3] to establish the diagnosis. Moreover, in 2021 the Chandos House meeting of the Alport Variant Collaborative extended the indications for screening for pathogenic variants in *COL4A3-5* genes beyond the classical Alport phenotype, to include persistent proteinuria, steroid-resistant nephrotic syndrome, FSGS, familial IgA glomerulonephritis and ESRD without an obvious cause. In these cases, genetic testing for AS diagnosis should precede renal biopsy. When a causative variant is confirmed, this procedure may be unnecessary [20]. 

AS disorders present a risk for the development of progressive kidney disease that can be delayed or possibly prevented by the timely initiation of therapy [1,21]. Although current treatment does not provide a cure, it can delay progression of Alport nephropathy, especially if initiated before there is a reduction in GFR [13]. Based on the European Alport Registry, it was found that ramipril can delay the onset of ESRD by 3 years in AS patients with CKD stages 3 and 4. Angiotensin-converting enzyme inhibitors (ACEi) can also delay CKD onset for 18 years in AS patients treated at the emergence of proteinuria [22]. These findings were corroborated by the EARLY PROTECT study. This study showed that ramipril decreased the risk of disease progression (HR 0.51, CI 0.12–2.20) and diminished albuminuria and the decline in GFR [5]. Moreover, when proteinuria is already established, ACEi are not as effective in delaying disease progression [21,22]. 

Based on these studies, it is recommended to initiate treatment at the time of diagnosis in males with X-LAS and in males and females with ARAS with more than 24 months of age and at the onset of microalbuminuria in females with XLAS and in males and females with ADAS [23]. In the case of ESRD, the only treatment options are dialysis and kidney transplantation [24]. In addition to ACEi, in all cases, a healthy lifestyle should be promoted—exercise, moderation in meat protein and salt intake, maintaining a body mass index less than 25 kg/m^2^; strict blood pressure control and no smoking are also recommended [24]. Paricalcitol and statins showed, in animal models, a nephroprotective effect and can be initiated as part of treatment recommendations in the case of secondary hyperparathyroidism and elevated cholesterol, respectively [24]. In a series of five pediatric patients (mean age 10.4 years, with eGFR > 60 mL/min/1.73 m^2^), the use of dapaglifozin reduced proteinuria in 22% by 12 weeks and was well tolerated [25]. An observational case series showed that Sodium-Glucose co-Transporter-2 (SGLT2) inhibitors were well tolerated and reduced albuminuria [26]. An International Panel agreed that SGLT2i seems a promising add-on therapy in AS patients at risk of progression [24]. 

In the last few years, alongside with the studies on early therapies, several biomarkers of renal damage in AS have been proposed. A search on PubMed on Alport Syndrome biomarkers in animal and human studies, published between 2010 and 2022, was conducted using keywords “Alport”, “COL4A”, “Biomarkers”. Additionally, we search for additional publications in the references of the selected articles. A section of novel biomarkers was also included due to the increasing development of new technologies. 

## 2. Biomarkers

In clinical practice, GFR estimation (based on serum creatinine) and albuminuria are widely used for renal disease diagnosis and prognosis. Estimated GFR (eGFR) correlates with the degree of kidney function, while albuminuria indicates the presence of glomerular damage. However, albuminuria and serum creatinine only increase when significant kidney damage is advanced and renal function has been lost [27,28,29]. 

In AS, as in other CKD etiologies, conventional biomarkers of renal damage increase relatively late. Moreover, there is evidence that the prompt initiation of ACEi therapy is crucial to change the natural course of this disease. Considering these facts, the identification of reliable and validated biomarker(s), defined according to the National Institutes of Health Biomarkers Definitions Working Group as “a characteristic that is objectively measured and evaluated as an indicator of normal biological processes, pathogenic processes, or pharmacologic responses to a therapeutic intervention” is important [30] to delay AS progression and maximize the effects of therapy.

The studies that have been performed in this field were essentially related to biomarkers that reflect the underlying pathophysiological process of AS renal damage. The studies performed including AS patients are summarized in Table 1.

### 2.1. Biomarkers of Glomerular Lesions

#### 2.1.1. Podocyturia

Podocyte depletion, as in other glomerular diseases, occurs in AS and precedes proteinuria. It is an important mechanism of progression of the disease, as it leads to glomerular tuft instability, glomerulosclerosis, and ultimately to ESRD [35,36,37]. Moreover, podocyturia is irreversible, and any attempt to reduce it, particularly in the early phases of glomerulopathy, is important to limit the progression of the disease [36,37]. Podocyte depletion can be noninvasively monitored through measurement of urine pellet podocyte-specific mRNAs, such as podocin [37].

In a study that included 358 patients with renal diseases and 291 controls, an independent cohort of 10 AS patients showed a 23-fold increase in urinary podocin mRNA levels [37]. 

In a cohort of 95 AS X-linked and ARAS patients, podocyte detachment rate (measured by podocin mRNA levels in urine pellets, expressed either per creatinine or 24 h excretion) was significantly increased compared to controls and before the onset of proteinuria or albuminuria. In the same study, the analysis of data from 41 archived biopsies of X-linked AS and ARAS patients showed that the average rate of podocyte loss in AS patients was 26 podocytes per year versus 2.3 in controls, representing an 11-fold increase in urine podocyte detachment rate. In addition, reduction in podocyte number and density in biopsies was correlated with proteinuria, glomerulosclerosis, and reduced renal function [12].

#### 2.1.2. Vascular Endothelial Growth Factor A

Vascular endothelial growth factor A (VEGFA) has an important role in preserving GFB function. Its over-expression as well as its down-regulation can cause GFB lesions and consequently proteinuria. Its main receptor—Vascular endothelial growth factor receptor 2 (VEGFR2)—mediates biological functions of VEFGA. After VEFGFR2 phosphorylation (p-VEFGR2), it can bind to the intracellular domain of nephrin, promoting its phosphorylation. The phosphorylated nephrin (p-nephrin) intervenes in podocyte-actin cytoskeleton dynamics and podocyte intercellular junction formation. Wang et al. (2015) found that VEGFA was accumulated in the GBM of AS patients. In addition, the expression of VEGFA and the levels of p-VEGFR2 and p-nephrin in glomeruli were increased and positively correlated with the amount of proteinuria in AS patients. Differently, expression of synaptopodin and nephrin were decreased and were negatively correlated with the degree of proteinuria in this cohort [31]. 

### 2.2. Biomarker of Tubular Lesion

#### Epidermal Growth Factor

Epidermal growth factor (EGF) is a peptide growth factor produced by the renal tubules that confers protection from kidney injury [27,28,32]. 

Urinary EGF (uEGF) has been independently associated with risk of progression of renal disease, as a marker of tubular damage and interstitial fibrosis [27,32]. Higher uEGF levels were associated with rapid decline of GFR and higher uEGF/creatinine ratio [28]. Li et al. (2018) compared uEGF normalized to urine creatinine (uEGF/Cr) in 115 AS pediatric patients with 146 healthy children. They found that uEGF/Cr decreases significantly faster with age in AS patients. Cross-sectionally, lower uEGF/Cr was accompanied by low GFR but not with 24 h proteinuria. In 38 patients with longitudinal follow-up, a significant inverse correlation was observed between uEGF/Cr and eGFR slope (r = 0.58, *p* < 0.001). Patients with lower uEGF/Cr were at increased risk of CKD progression [32].

### 2.3. Biomarkers of Inflammation

#### 2.3.1. Transforming Growth Factor Beta 1 and High Motility Group Box 1

Transforming Growth Factor Beta 1 (TGF-β1) is a cytokine secreted in the extracellular space that is involved in the control of proliferation and cell differentiation. It has a pro-fibrotic effect on glomeruli and tubulointerstitial area, as it promotes synthesis and suppresses the degradation of matrix proteins. TGF-β1 can also induce apoptosis of endothelial cells, podocytes, and tubular epithelial cells [33,38]. It has also been reported that TGF-β1 is significantly elevated in children with CKD in comparison to healthy children [38]. In a study that included 10 X-linked male dogs and five controls aimed at examining the evolution of renal damage in serial biopsies and the expression of some molecules potentially involved in the pathogenesis of the disease, at baseline (T0) TGF-β, connective tissue growth factor and platelet-derived growth factor α were overexpressed in X-linked dogs compared with controls [39]. Another important finding was that, at T0, all X-linked dogs had proteinuria despite the presence of negligible light microscopic changes [39]. 

High Motility Group Box 1 (HMGB1) intervenes in innate immunity with actions on cellular signaling and inflammatory responses [33]. 

In a case-control study that compared 10 healthy subjects to 11 AS pediatric patients with normal values of albuminuria and proteinuria without CKD, AS patients presented higher levels of serum and urinary HMGB1 and TGF-β1, suggesting that subclinical pro-fibrotic and inflammatory processes are triggered before the appearance of proteinuria [33]. 

#### 2.3.2. Urinary Monocyte Chemoattractant Protein-1

Monocyte Chemoattractant Proteina-1 (MCP-1) is a powerful regulator of monocyte migration and infiltration. It identifies patients at risk for renal fibrosis and hence worse renal outcomes [40]. 

In a case-control study including 76 patients, mainly with X-linked AS, with normal renal function, 40 healthy adults and 11 pediatric controls, the concentration of a set of 18 proteins in urine was determined. It was found that urinary MCP-1 (uMCP-1)/Creatinine (Cr) levels at baseline were significantly higher in AS patients. In a subgroup of 28 patients that were followed for 5 years, baseline urinary uMCP-1/Cr was negatively correlated with the slope of eGFR decline in 19 male patients, while no correlation was found in 9 female AS patients [34]. 

### 2.4. Novel Biomarkers

#### 2.4.1. Proteomics

Proteomics give information about all proteins that are present in different samples (blood, urine, tissues); it is also known as protein expression profile or protein signature.

A study that used the proteomics approach for the identification of different protein expression in glomeruli using *Col4a3*-null mice and wild-type mice found that vimentin (a podocyte protein) was upregulated 2.5 fold in AS mice glomeruli compared to wild-type mice glomeruli. In addition, integrin (main receptors for GBM type IV collagen and laminin) expression, namely integrin α1 and integrin α3, was increased. These findings suggest that cell signaling, cell shape and cellular adhesion to the GBM could be compromised in AS [41]. The proteomics approach was used in another study that used a *Col4a3*-null mice model and wild-type mice to find serum pre-clinical alterations. The results showed that the serum composition was dependent on age, i.e., stage of disease, and therapy. Initially, the main proteins found were to be related to extracellular matrix remodeling, cell damage and the production of acute phase proteins, such as kininogens, retinol binding domain containing protein 4, antithrombin, serum amyloid P, plasminogen, apo-lipoproteins (Apo) AI, AIV, CIII, and E, sub-fractions of transferrin, 2-glycoprotein 1, and the chemoreceptor vomeronasal type 2 receptor. Importantly, the amount of collagen α1(I) (COL(I)A1), isoform 3 of sulfhydryl oxidase 1, leucine rich HEV glycoprotein, and a special variant of inter-alpha-trypsin inhibitor were higher in AS mice. In later stages, renal filtration failure and systemic acute phase reaction determine the composition of the serum, such as haptoglobin, Apo B, and 2-glycoprotein 1 and peptides related to transferrin [42].

Using animal models of *COL4a3-/-* and *Col4a5-/-* mice, proteomics analysis of renal tissue found an overall reduction in basement membrane proteins in the glomerular matrix (laminins, type IV collagen, type XVIII collagen) and an overall increase in interstitial matrix proteins (collagens I, III, VI, and XV; fibrinogens and nephronectin) compared to wild-type mice [43]. 

#### 2.4.2. MicroRNA

MicroRNAs (miRNAs, miRs) are small, non-coding RNAs that post-transcriptionally regulate gene expression. The complex miRNA-mRNA influences various physiological and pathological processes [44].

In an AS family, 30 known miRNAs were identified and shown to be significantly differently expressed in AS patients and family controls. Nineteen miRNAs were up-regulated, and eleven were down-regulated. Hsa-miR-3117-3p was the most significantly up-regulated expressed miRNA, with a fold-change of 7.785, and hhsa-miR-544b was the most significantly down-regulated expressed miRNA, with a fold-change of −9.085 [45]. 

One study applied small RNA sequencing to identify different miRNA profiles in five dogs with X-linked AS and four age-matched controls. Five microRNAs—miR-21, miR-146b, miR-802, miR-142 and miR-147—were consistently upregulated in affected dogs, suggesting that they can be involved in the regulation of important pathways of CKD progression in the models used [44]. 

Another pre-clinical study showed an abnormal renal tissue expression of miR-21 in dogs suffering from X-linked hereditary nephropathy. Kidney biopsies were performed at several time points during disease progression, including ESKD. In XLHN dogs, kidney miR-21 began to be significantly upregulated at the time point at which serum creatinine was increased. Moreover, miR-21 expression was positively correlated with the urine protein:urine creatinine ratio, serum creatinine concentration, and histologic lesions (glomerular damage, tubular damage, chronic inflammation and fibrosis). These findings suggest that miR-21 may have an important role in disease progression [46].

Another study that evaluated miR-21 expression in renal tissue from 27 patients with AS found that miR-21 expression was significantly elevated in kidney specimens from those patients as compared to normal controls. Moreover, high renal miR-21 expression positively correlated with 24 h urine protein, serum blood urea nitrogen, serum creatinine and the severity of kidney pathology. On histological evaluation, increased levels of miR-21 were localized to damaged tubular epithelial cells and glomeruli [47]. 

## 3. Conclusions and Perspectives

AS is an important cause of monogenic renal disease that remains underdiagnosed [48,49,50,51]. Genetic studies have allowed the recognition that autosomal dominant forms are more prevalent than previously described and that the genotype has an important role in determining when to start treatment and inferring prognosis [13,21,22,52]. Although ACEi-based current treatment confers significant renal protection—if promptly started, it can delay in decades the need of a renal substitute therapy—it does not offer a cure [21,22,52]. 

Although many advances have been observed in pathogenesis, genetic and treatment fields, many areas are still undefined, such as the selection of the appropriate moment to start therapy in ADAS cases and X-linked females [24], how to monitor treatment efficacy and the impact of new therapy approaches [24,53]. 

Urinary and serum biomarkers of AS, contrary to tissue biomarkers, are non-invasive and therefore can be very promising to answer these questions. 

The traditional biomarkers, such as albuminuria and elevation of serum creatinine, are not early enough to reflect the onset of renal damage. Thus, the treatment guided by these parameters could result in an important time lapse and loss of opportunity to improve prognosis [27,28,29]. 

**Figure 2:**
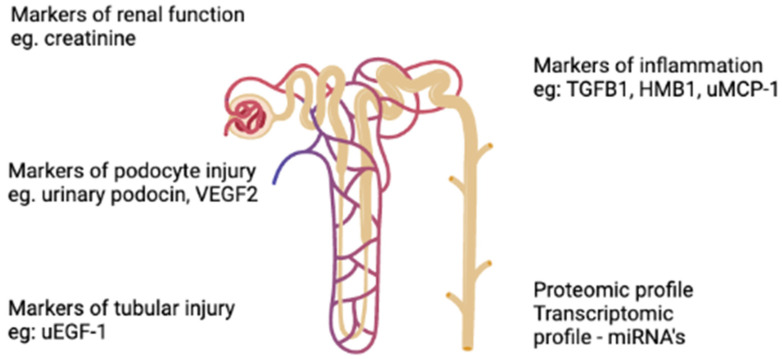
Biomarkers of Alport Syndrome according to localization and/or site production.

Urinary podocin and VEGF-A are important markers of podocyte injury and can be elevated even before the onset of proteinuria and seem to be promising to detect initial renal damage. Moreover, inflammatory biomarkers, such as TGFβ-1, HMGB-1 and uMCP-1 seem to be related to the risk of rapid progression of the disease and also can be elevated before the onset of proteinuria. uEGF and miRNA-21 are elevated when renal damage is established. The biomarkers of AS according to the anatomic localization and/or site of production are described in Figure 2. 

At this point, it is not possible to defined the “perfect” biomarker of AS. A panel of biomarkers is a better approach to determine renal damage in AS, as in other forms of CKD, than a single biomarker, unsurprisingly, given the multiple pathophysiological pathways involved in AS [29]. There also other molecules involved in the disease pathways that haven not been explored as biomarkers for AS, such as ET-1, NF-ĸB and metalloproteinases. 

Based on this, the demand for the identification of new biomarkers that can reflect kidney damage and help in the decision to intervene is urgent. In addition, new biomarkers could contribute to therapeutic efficacy monitoring and help in developing new drugs that directly intervene in the disease pathways, such as anti-microRNA-21 and anti-endothelin receptors, as recently reported in [24,53]. 

## Figures and Tables

**Table 1:** Biomarkers identified in Alport Syndrome Patients.

Year	Study Type	Study Population	Biomarker	Study Outcomes	Reference
**2015**	Cross-sectional	25 AS patients and 11 controls	Tissue VEGFA, synaptopodin and nephrin	Accumulation of VEFGA in the GBM was increased in AS patients. This may lead to activating VEGFA-VEGFR2 and nephrin signaling pathways, resulting in podocyte injury and proteinuria.	[31]
**2017**	Cross-sectional	2 cohorts: urine samples—95 X-linked and ARAS patients vs. 38 controls and biopsy samples: 41 X-linked and ARAS patients vs. 20 controls	Urine podocin mRNA Podocyte count in renal biopsy	Podocyte detachment rate (measured by podocin mRNA in urine pellets expressed either per creatinine or 24 h excretion) was significantly increased 11-fold above control, and before increased proteinuria or albuminuria. The average rate of podocyte loss in AS patients was 26 podocytes per year versus 2.3 in controls.	[12]
**2018**	Cross-sectional Longitudinal	117 AS pediatric patients and 146 controls 38 AS pediatric patients followed for an average period of 31 months	uEGF	uEGF/Cr decreases with age in AS patients with a significantly faster rate than in healthy children. Lower uEGF/Cr is significantly correlated with eGFR slope.	[32]
**2020**	Cross-sectional Longitudinal	11 AS pediatric patients and 10 controls	Urinary and serum HMGB1 Urinary and serum TGF-β	AS had significantly higher levels of serum and urinary HMGB1; the same trend was observed for TGF-β1.	[33]
**2022**	Cross-sectional Longitudinal	76 AS patients and 51 controls 28 patients FU 5 years	Urinary MCP-1	uMCP-1 levels were significantly higher in AS patients. In male AS patients, baseline uMCP-1 was negatively correlated with the slope of eGFR decline.	[34]

## Abbreviations

AS	Alport Syndrome
CKD	Chronic Kidney Disease
GBM	Glomerular basement membrane
GFB	Glomerular filtration barrier
ESRD	End-stage renal disease
ET-1	Endothelin-1
ETARs	Endothelin A receptors
FAK	Focal adhesion kinase
NF-ĸB	Nuclear factor kappa B
GFR	Glomerular filtration rate
ARAS	Autosomal Recessive Alport Syndrome
ADAS	Autosomal Dominant Alport Syndrome
FSGS	Focal segmental glomerulosclerosis
EM	Electronic microscopy
ACEi	Angiotensin-converting enzyme inhibitor
SGLT2i	Sodium-Glucose co-Transporter- 2 inhibitors
eGFR	Glomerular filtration rate estimation
VEGFA	Vascular endothelial growth factor A
VEGFR2	Vascular endothelial growth factor receptor 2
p-VEFGR2	Vascular endothelial growth factor receptor 2 phosphorylated
p-nephrin	Phosphorylated nephrin
EGF	Epidermal growth factor
uEGF	Urinary epidermal growth factor
TGF-β1	Transforming Growth Factor Beta 1
HMGB1	High Motility Group Box 1
MCP-1	Monocyte Chemoattractant Proteina-1
uMCP-1	Urinary Monocyte Chemoattractant Proteina-1
